# Cannabis, Cannabinoids, and the Endocannabinoid System—Is there Therapeutic Potential for Inflammatory Bowel Disease?

**DOI:** 10.1093/ecco-jcc/jjy185

**Published:** 2018-11-12

**Authors:** Tim Ambrose, Alison Simmons

**Affiliations:** 1 Translational Gastroenterology Unit, John Radcliffe Hospital, Oxford, UK; 2 MRC Human Immunology Unit, John Radcliffe Hospital, Oxford, UK

**Keywords:** Inflammatory bowel disease, cannabis, cannabinoids

## Abstract

*Cannabis sativa* and its extracts have been used for centuries, both medicinally and recreationally. There is accumulating evidence that exogenous cannabis and related cannabinoids improve symptoms associated with inflammatory bowel disease [IBD], such as pain, loss of appetite, and diarrhoea. *In vivo*, exocannabinoids have been demonstrated to improve colitis, mainly in chemical models. Exocannabinoids signal through the endocannabinoid system, an increasingly understood network of endogenous lipid ligands and their receptors, together with a number of synthetic and degradative enzymes and the resulting products. Modulating the endocannabinoid system using pharmacological receptor agonists, genetic knockout models, or inhibition of degradative enzymes have largely shown improvements in colitis *in vivo*. Despite these promising experimental results, this has not translated into meaningful benefits for human IBD in the few clinical trials which have been conducted to date, the largest study being limited by poor medication tolerance due to the Δ^9^-tetrahydrocannabinol component. This review article synthesises the current literature surrounding the modulation of the endocannabinoid system and administration of exocannabinoids in experimental and human IBD. Findings of clinical surveys and studies of cannabis use in IBD are summarised. Discrepancies in the literature are highlighted together with identifying novel areas of interest.

## 1. Introduction

The use of cannabis, whether for medicinal or recreational purposes, dates back to ancient civilisation, featuring in Chinese medicine almost 5000 years ago and also described in Egyptian, Greek, Indian, and Middle Eastern cultures.^1^ Within Western medicine, the earliest work was performed by William O’Shaughnessy in the mid 1800s.^2^ He defined effects of Indian hemp on healthy animals and in human cases of rheumatism, hydrophobia, cholera, tetanus, and infantile convulsions.^3^ Despite medicinal use for millennia, work continues to try to understand the mechanistic role of cannabinoids in gastrointestinal disease, including human inflammatory bowel disease and animal models of intestinal inflammation.

## 2. The endocannabinoid system

The major active ingredient of *Cannabis sativa*, and that which causes the psychotropic effects, is Δ^9^-tetrahydrocannabinol [THC], and was only isolated from the plant in 1964^4^ when technology advanced to allow isolation and characterisation of individual components of mixtures. However, the plant contains up to 100 cannabinoid constituents including cannabidiol [CBD].^5^ For some years after the identification of THC, the potential target[s] of action of cannabinoids remained elusive.

The discovery of cannabinoid receptor 1 [CB1] in the 1980s as both an abundant G-protein coupled receptor [GPR] in the human brain,^6^ and also at lower levels in immune cells,^7–12^ was followed by cannabinoid receptor 2 [CB2]. This was identified in the human HL60 promyelocytic leukaemia cell line and has 44% homology to CB1.^13^ CB2 has been described in human immune cell subsets,^8,11,12^ particularly those of a myeloid lineage.^14^ Since the identification of CB1/2, other putative cannabinoid receptors have been identified, including GPR18,^15^ GPR55,^16^ GPR119,^17^ transient receptor potential channels,^18,19^ and peroxisome proliferator-activated receptors.^19^

Subsequently, endogenous ligands for these receptors, through which THC exerts its actions, were identified. Anandamide [AEA] is a partial agonist of CB1 and CB2, with 2-arachidonoylglycerol [2AG], a monoacylglycerol, acting as a full agonist at both these receptors although with greater potency for CB1 than CB2.^19,20^ Other bioactive lipids related to these classical endocannabinoids have been described, including N-acyl-ethanolamines (palmitoylethanolamine [PEA], oleoylethanolamine [OEA]) and other monoacylglycerols (2-oleoylglycerol [2OG], 2-palmitoylglycerol [2PG]). They may also bind to other cannabinoid-related receptors^21^ to exert actions independent of CB1/2. They may act to modulate 2AG signalling through an ‘entourage effect’,^22,23^ although whether this is to potentiate or inhibit is dependent on the experimental model used. Interestingly, gut microbes have been shown to produce lipid ligands with similarities to the wider endocannabinoids to signal through GPRs, including GPR119, to modulate host physiology particularly with glucose handling.^24^

2AG is synthesised from the action of diacylglycerol lipases [DAGL] α and β on diacylglycerols [DAGs] containing the arachidonate moiety. Metabolism of 2AG may proceed through hydrolysis, oxidation by COX2 or LOX enzymes, or epoxidation by components of the cytochrome P450 system,^25,26^ to produce arachidonic acid and eicosanoids [prostaglandins and leukotrienes]. In the murine brain, monoacylglycerol lipase [MGLL] accounts for 85% of 2AG hydrolytic activity, with further contributions by αβ-hydrolase domain 6 [ABHD6] and ABHD12.^27^ Fatty acid amide hydrolase [FAAH], the chief hydrolytic enzyme for AEA,^28^ also contributes slightly to 2AG hydrolysis. The contribution of MGLL to prostaglandin production downstream of 2AG appears to vary by tissue type—MGLL regulates prostaglandin production in murine liver and lung, and PLA2G4A in gut and spleen, with contributions from both enzymes in brain tissue.^29^ MGLL has also been demonstrated to hydrolyse prostaglandin glycerol esters [produced from 2AG by COX2 oxidation] and so may have more substrates than just monoacylglycerols.^30^

The cannabinoid receptors, together with their endogenous ligands and synthetic/degradative enzymes, form the endocannabinoid system [ECS] through which THC and other exocannabinoids act.

## 3. Inflammatory bowel disease

IBD can be classified into Crohn’s disease [CD] and ulcerative colitis [UC], based on characteristic clinical, radiological, endoscopic, and histological features. Although incompletely understood, the aetiology is thought to represent a complex interaction between genetic background, intestinal microbiota, environmental factors, and host immune response, resulting in persistent and dysregulated inflammation.

The incidence of IBD has increased in Western populations^31,32^ although it may be plateauing.^33^ However, this is not the case in the Asia-Pacific region where both incidence and prevalence are increasing.^34^ The increased incidence cannot be accounted for by substantial changes in host genetics, with environmental factors likely to be of critical importance [reviewed in ^35^ and briefly summarised in Table 1]. Although there has been an explosion in therapies for IBD, there remains an unmet clinical need for novel therapies and improved understanding of disease biology.

**Table 1.** Effect of environmental factors on rates of IBD.

Factor	General effect on rates of IBD
**South to North migration**	Increase
**East to West migration**	Increase
**Smoking**	Reduction [UC]; increase [CD]
**Appendectomy**	Reduction [UC]; increase [CD]
**Antibiotics in childhood**	Increase [Western populations]; reduction [Asia]
**Improved hygiene/sanitation**	Increase
**COX inhibition**	Increase [NSAIDs, aspirin]; reduction [COX2-inhibitors]
**Reduced dietary fibre**	Increase [CD]
**High ω-6:ω-3 PUFA**	Increase
**Vitamin D deficiency**	Increase
**Stress**	Increase
**Increased physical activity**	Reduction

COX, cyclo-oxygenase; PUFA, polyunsaturated fatty acids; NSAID, non steroidal anti-inflammatory drug; CD, Crohn’s disease; UC, ulcerative colitis.

Data extracted from reference 35.

Genetic association studies have identified approximately 200 loci associated with IBD, with many shared between CD and UC.^36,37^ Differences in susceptibility alleles exist between ethnic backgrounds with, for instance, NOD2 and IL23R variants over-represented in European populations compared with East Asian populations.^34,37^ More recently, using high-density genotyping, 45 variants have been fine-mapped as potentially causal for IBD,^38^ and this approach may aid better understanding of disease mechanisms. Grouping of susceptibility loci using ontology pathway analysis has highlighted broad mechanisms of relevance in the immunological response in IBD, and this has informed subsequent study. This includes autophagy [ATG16L1, IRGM, LRRK2], endoplasmic reticulum stress [XBP1], innate immune cell sensing [NOD2], T cell tolerance [IL10, IL10R], IL23-pathways [IL23R, IL12B], lymphocyte trafficking [CCL7, IL8], and epithelial barrier function [MUC1, MUC3].^39–42^ Another development in the field of IBD genetics is evidence that genetic associations may be related to disease prognosis in addition to, or instead of, disease susceptibility.^43^ This might provide a novel avenue to stratify patients and target therapies accordingly.

## 4. Single nucleotide polymorphisms in ECS components in IBD

Single nucleotide polymorphisms [SNPs] in some ECS genes have been investigated in human IBD [Table 2]. The Q63R mutation in the CNR2 gene, encoding CB2, impairs endocannabinoid-induced inhibition of T cell proliferation.^44^ In an Italian paediatric IBD cohort, this mutation was associated with a more severe disease phenotype and shorter time to relapse for UC,^45^ but these were not replicated in a Turkish cohort of adult patients.^46^ This may be due to either age or ethnic differences between patients. The G1359A mutation in CNR1, encoding CB1, has been shown to have a lower prevalence in patients with UC than in controls, and to be associated with lower body mass index [BMI] and later age of onset in CD.^47^ The C385A substitution in FAAH results in reduced FAAH expression, but there are no differences in genotype prevalence in IBD.^48,49^ However, there is a possible association of the AA genotype with a penetrating phenotype and increased extra-intestinal manifestations in CD, and earlier age of onset in UC.^48^ Interestingly, the FAAH mutation is more prevalent in patients with diarrhoea-predominant or mixed-picture irritable bowel syndrome,^50^ suggesting effects on motility, secretion or pain perception.

**Table 2.** Single nucleotide polymorphisms of components of the ECS studied in human IBD.

		Genotype			
**FAAH C385A**		**CC**	**CA**	**AA**	**Associations**
**Storr 2008**	CD [*n* = 202]	67.3%	31.2%	1.5%	No differences in prevalence between groups. Phenotype not assessed in this study
	Controls [*n* = 206]	63.6%	35.0%	1.5%	
**Storr 2009**	CD [*n* = 435]	65.8%	30.1%	4.1%	No differences in prevalence between groups. AA associated with more EIMs and penetrating phenotype in CD; earlier age of onset in UC.
	UC [*n* = 167]	65.3%	32.9%	1.8%	
	Controls [*n* = 406]	61.6%	34.5%	3.9%	
**CNR1 G1359A**		**GG**	**GA**	**AA**	
**Storr 2010**	CD [*n* = 216]	53.3%	39.8%	6.9%	Lower prevalence of AA in UC versus controls. AA associated with lower body mass index and later age of onset of CD.
	UC [*n* = 166]	58.4%	38.0%	3.6%	
	Controls [*n* = 197]	52.3%	37.0%	10.7%	
**CNR2 Q63R**		**QQ**	**QR**	**RR**	
**Yonal 2014**	CD [*n* = 101]	10.9%	38.6%	50.5%	No differences in prevalence or phenotype in this study.
	UC [*n* = 101]	6.9%	43.6%	49.5%	
	Controls [*n* = 101]	11.9%	37.6%	50.5%	
**Striscuglui 2016**	CD [*n* = 112]	2.7%	48.2%	49.1%	Paediatric cohort. RR genotype more prevalent in IBD than controls and associated with more severe disease activity at diagnosis. In UC, associated with higher risk of relapse.
	UC [*n* = 105]	18.1%	38.1%	43.8%	
	Controls [*n* = 600]	16.0%	51.7%	32.3%	

Prevalence of genotypes are displayed alongside associations with disease phenotype. Data extracted from references 45–49.

IBD, inflammatory bowel disease; CD, Crohn’s disease; UC, ulcerative colitis; ECS, endocannabinoid system; EIM, extra-intestinal manifestations.

## 5. ECS tone in IBD

**Figure 1.**
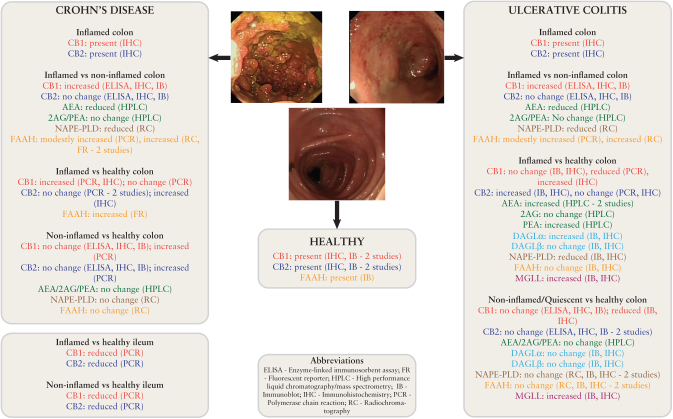
A summary of existing studies of the endocannabinoid tone in human ileum and colon in health and IBD. Studies of the ECS in human IBD have yielded conflicting results dependent on the technique used, the site of sampling [ileum vs colon], and the comparison used [inflamed vs non-inflamed vs healthy]. Only one study has assessed the synthetic [DAGL] and key hydrolytic [MGLL] enzyme involved in 2AG metabolism. No studies have examined the presence of ABHD6 or 12. Data extracted from references 48, 51–59. IBD, inflammatory bowel disease; ECS, endocannabinoid system.

There is disagreement on the tone of the ECS between studies of human IBD [summarised in Figure 1]. CB1 has been identified in the colonic epithelium [particularly crypts^51^], some plasma cells of the lamina propria, smooth muscle, and the submucosal myenteric plexus.^52^ Conversely, CB2 localises in the absorptive and goblet cells of the epithelium, Paneth cells, and some subepithelial macrophages and plasma cells.^51,52^ CB2 was expressed at slightly higher levels than CB1 in one study.^53^ The effect of inflammation on expression of CB1 and CB2 is less clear. Increases in both CB1 and 2 in both CD and ‘acute phase IBD’ [a combination of UC and IBD-unclassified]^52^ have been observed. The authors of this study suggest that the changes in CB1 may be an effect of goblet cell depletion in epithelial architecture rather than a true increase in protein abundance. In other work, increases in either CB1^54,55^ or alternatively in CB2^51^ alone have been observed. A recent study of CB1 and CB2 gene expression in Crohn’s disease, the largest to date, demonstrated consistent detection of expression albeit at low levels in inflamed, non-inflamed, and healthy samples. A difference in expression patterns was seen between disease affecting the ileum, where both CB1 and 2 were reduced in inflamed/non-inflamed samples, and colon, where an increase was seen in CB1 and 2 in the non-inflamed but not inflamed samples.^56^

Similar controversy exists for levels of endocannabinoids themselves. AEA has been shown to be increased in inflamed samples from UC patients,^57,58^ yet reduced in another study.^54^ It is more plausible that AEA levels are indeed decreased in inflammation as it has been shown that there is reduced expression of the synthetic enzyme NAPE-PLD and increased expression of the hydrolytic enzyme FAAH in colonic inflammation.^51,54,59,60^ PEA is increased in inflammation in one study^58^ but does not change in another,^54^ and the two studies assessing 2AG levels both demonstrate no change in inflammation.^54,57^

To date, only one study has attempted to define levels of MGLL and the DAGL enzymes in human IBD.^51^ MGLL was localised to the central portion of epithelial cells, polymorphonuclear cells of the lamina propria, and the myenteric plexus, and was shown to be increased in inflammation. Previously MGLL has been shown to be widely distributed in the rat intestine, increasing in expression from duodenum to distal colon.^61^ DAGLα was similarly increased in inflammation, but no difference was seen in DAGLβ.

There are a multitude of potential explanations for the differences between these studies. In particular, the patients studied were not always well phenotyped for disease severity and extent, and were frequently grouped together. Furthermore, the potential role of medications such as systemic immunosuppression in altering expression levels was not explored. In many studies, comparisons were made between inflamed mucosa in an IBD patient and healthy mucosa from a different person. ECS tone has been shown to be affected by diurnal variation^62^ and ethnicity,^63^ not to mention the likely confounder of any comorbidities such as irritable bowel syndrome, in which the involvement of the ECS is well documented.^64^ Definitive annotation of ECS component distribution in the intestine will benefit from development of more sensitive and specific antibodies.

Interestingly, CBD reduces iNOS and S100B expression in cultured colonic biopsies from patients with UC, indicating effects on enteric glial cell activation. These effects were dependent on PPARγ, being abrogated by a PPARγ antagonist.^65^ Furthermore, fractions of THC have recently been extracted and assayed for effects on colonic IL-6/8 in *ex vivo* biopsies, with the greatest benefit seen with Δ^9^-tetrahydrocannabinolic acid [THCA] which does not have psychoactive effects.^66^ THCA has not yet been used in clinical trials.

## 6. *In vivo* studies

### 6.1. Cannabinoid receptors

One of the earliest studies of the effect of cannabinoid receptor modulation identified that CB1^-/-^ mice exhibited more severe colitis following either dinitrobenzene sulphonic acid [DNBS] or dextran sulphate sodium [DSS] administration.^67^ Subsequently this finding was confirmed in trinitrobenzene sulphonic acid [TNBS] colitis and extended to demonstrate that CB2^-/-^ and CB1^-/-^/CB2^-/-^ double knockout mice also display worsened colitis.^68^ Although not reaching statistical significance, there was an additive effect of double knockout on macroscopic colonic inflammation but not histological inflammation. The effects of CB2^-/-^ have also been confirmed in DSS colitis. The authors also demonstrated that murine peritoneal macrophages stimulated *ex vivo* with lipopolysaccharide [LPS]/DSS upregulated components of the NLRP3 inflammasome, that this was exacerbated in CB2^-/-^ cells, and was improved by the CB2 agonist HU308.^69^

The findings of genetic knockout studies have largely been confirmed by use of CB agonists/antagonists. DNBS colitis is worsened by prophylactic administration of the CB1 antagonist SR141716A,^67^ with DSS and oil-of-mustard colitis improved by prophylactic ACEA, a CB1 agonist.^70^ Similarly, the CB2 agonists JWH133,^70^ HU308,^69^ or ALICB459^71^ are beneficial in ameliorating chemical colitis models when administered prophylactically. ALICB459 is particularly appealing from a translational medicine perspective, as it was effective with oral rather than intraperitoneal administration. CB2 agonists improve, and CB2 antagonists worsen, chemical colitis when administered therapeutically, and that this is CB2-dependent.^72–74^

Although most studies have used chemical colitis models, JWH133 ameliorates colitis in the IL10^-/-^ model where mice develop spontaneous colitis by 12 weeks of age.^73^ GP-1a, purported to be a CB2 agonist, improves ileitis when administered retro-orbitally in the TNF^ΔARE^ model of Crohn’s-like ileitis. However, recent evidence would suggest that this compound may in fact be an inverse agonist of both CB1 and CB2 *in vitro*,^75^ and so it remains to be confirmed whether CB2 agonism or inverse agonism is effective here.

Complementing the results from double knockout studies, use of less selective agonists also display benefits in colitis. WIN55,212 is an agonist of both CB1 and CB2 and ameliorates colitis whether used prophylactically or therapeutically in TNBS and DSS models.^76–78^ AM841 [CB1 agonist] improves colitis in a cannabinoid receptor-dependent manner, with the effect lost in CB1^-/-^ and CB2^-/-^ mice.^79^ The same is true for the CB1 agonist, HU210,^67^ which also has effects on sustaining intestinal barrier function in a TLR4-independent manner.^80^ Interestingly the effect of non-selective cannabinoid receptor activation may be through central rather than peripheral mechanisms. CB13 is an agonist of both CB1 and CB2, with poor penetration of the central nervous system.^81^ However, this compound was not effective in murine TNBS or DSS colitis when administered intraperitoneally, but was effective when injected intracerebroventricularly.^79^ Equally ineffective was the peripherally restricted, non-selective agonist, SAB378.^76^

Two studies have suggested interplay between cannabinoid signalling and p38 MAPK in the modulation of colitis severity. In the first,^77^ Mk2^-/-^ mice, who lack a downstream substrate of p38, exhibit a less severe colitis in response to DSS. This study also demonstrated that WIN55,212 impairs phosphorylation of p38 in response to DSS in both wild-type and Mk2^-/-^ mice. Subsequently a similar result has been obtained by using the p38 inhibitor SB203580.^78^ One mechanism therefore, by which colitis is ameliorated by cannabinoids, may be through effects on MAPK pathways.

### 6.2. Endocannabinoid lipid ligands

A recent study investigated rectal 2AG administration in chemical colitis.^82^ Here the authors used carbon nanotubes linked to 2AG, with the aim of reducing rapid hydrolysis and improving the overall pharmacological profile. A single dose administered rectally 2 days before the instillation of TNBS in rats, and then a second dose 8 days after instillation, resulted in improvement of colitis. No effect was seen of free 2AG or of the carbon nanotubes alone.

In addition to the effect of 2AG on colitis, intraperitoneal AEA improves TNBS colitis.^83^ More evidence exists for the role of PEA in colitis, possible acting by inhibiting the induction of angiogenesis that is usually seen in chemical colitis.^84^ Intraperitoneal injection of PEA is beneficial in both established chemical colitis and when administered prophylactically.^85^ The effects are dose-dependent and require PPARα but not PPARγ.^86^ This study demonstrated a reduction in TLR4 and S100B expression on enteric glial cells and a reduction in MAPK signalling, as potential mechanisms of action. The effect of PEA is also dependent upon CB2 and GPR55.^87^ Adelmidrol is a PEA analogue which is effective orally in established colitis in a PPARγ- but not PPARα-dependent manner, contrary to the earlier study.^88^

### 6.3. Hydrolytic enzymes

#### 6.3.1. MGLL

To date, only one study has assessed the role of MGLL inhibition in colitis.^89^ Rectal administration of TNBS was used to induce colitis, and the small molecule MGLL inhibitor JZL184 was administered prophylactically. Both macroscopic and microscopic colitis were ameliorated. This was associated with a reduction in mucosal and systemic pro-inflammatory cytokines such as IL-6, TNFα, and IL-12. MGLL inhibition also reduced LPS-induced endotoxaemia, which may suggest effects on mucosal barrier function. Certainly, THC and CBD have beneficial effects on intestinal permeability induced by EDTA in unstimulated Caco-2 [colonic carcinoma] cells.^90^ The effects of 2AG and AEA in this model were dependent on apical [worsened permeability] versus basolateral [improved permeability] administration. The same is true for JZL184 administration in unstimulated Caco-2 cells.^91^ When cytokines were administered to Caco-2 cells to mimic inflammation, JZL184 worsened permeability when applied apically either at the same time as the cytokines^92^ or after inflammation had been induced.^91^ The benefits of MGLL inhibition on TNBS colitis involved both CB1 and CB2, as inhibition of either abrogated the effects of JZL184.^89^ However the CB2 antagonist used, AM630, has recently been shown to have off-target effects,^75^ and so it would be useful to have data using alternative CB2 antagonists to confirm the role of this receptor in mediating the effects seen. It is also worth noting that the dose of JZL184 used here is high [32 mg/kg daily in divided doses] and is within the dose to desensitise CB1,^93^ although it is not clear whether this happens within the 3-day time frame used in this model.

#### 6.3.2. FAAH

Although only one *in vivo* study has been performed using MGLL inhibition, several studies have investigated FAAH inhibition. First, FAAH^-/-^ mice exhibit less severe chemical colitis.^67^ Use of pharmacological FAAH inhibitors corroborates the findings from the genetic study with amelioration of disease^49,57,94,95^ in a CB1- and CB2-dependent manner.^49^ A combined FAAH/COX inhibitor, ARN2508, was effective in a CB1- and PPARα-dependent manner.^96^ Inhibition using FAAH-II not only improved colitis and reduced pro-inflammatory cytokine production, but also impaired infiltration by immune cells and affected expression of micro-RNAs in mesenteric lymph nodes and Peyer’s patches of colitic mice^97^ as a potential mechanism of action. One study has suggested that therapeutic FAAH inhibition may have additional benefits over prophylactic administration, although this remains to be replicated.^98^ Despite several studies demonstrating a benefit of FAAH inhibition, findings are not unanimous. One study demonstrated that PF3845 is effective at ameliorating colitis in the TNBS model but not the DSS model.^99^ The beneficial effects on TNBS were not replicated by a second group.^85^

Inhibition of N-acylethanolamine-hydrolysing acid amidase [NAAA] results in increased levels of PEA and not AEA, and improvement in colitis.^85^ This corroborates the studies performed using PEA.

### 6.4. Other ECS targets

It is not well understood to what extent the non-CB1, non-CB2 cannabinoid receptors have roles in colitis. The atypical cannabinoid O-1602 is a derivative of CBD and is known to bind to GPR55. Although it exerts anti-inflammatory effects on both DSS and TNBS colitis, this is independent of GPR55 as genetic knockout of this receptor, and indeed of both CB1 and CB2, does not alter the effect of O-1602.^100^ It is not known therefore by which pathways this compound acts on in colitis. The GPR55 antagonist CID16020046 and GPR55^-/-^ mice exhibit less severe colitis in response to DSS or TNBS, contrary to genetic knockout or pharmacological inhibition of CB1/2.^101^ Therefore, whereas CB1/2 are likely to have anti-inflammatory effects, GPR55 triggers a pro-inflammatory cascade.

Inhibition of AEA reuptake using the endocannabinoid membrane transport inhibitor VDM11 has also been shown to improve experimental colitis.^49,57^

### 6.5. Exocannabinoids

Further to the studies modulating endocannabinoid levels, administration of exocannabinoids in experimental colitis has largely demonstrated similar benefit. Β-caryophyllene is available orally and acts via CB2 and PPARγ to limit colitis.^102^ Similarly, αβ-amyrin ameliorates both TNBS^103^ and DSS^104^ colitis and acts, at least in part, through cannabinoid receptors. The phytocannabinoid CBD has been most extensively studied. It reduces intestinal inflammation induced by LPS as measured by TNFα.^65^ A reduction in inducible nitric oxide synthase [iNOS] expression has been demonstrated by CBD which also reduces IL-1β and increases IL-10 levels.^105^ The effect of CBD is maintained when administered intraperitoneally or rectally but not orally.^106^ It has also been shown to potentiate the anti-inflammatory effects of THC in chemical colitis.^107^ Synthetic derivatives such as abnormal CBD ameliorate colitis independently of CB1/2 and possibly through GPR18,^108^ whereas the highly concentrated CBD, known as CBD botanical drug substance [a major ingredient of nabiximols], was effective orally and intraperitoneally unlike pure CBD.^109^ Reference has been made to benefit of CBD in the IL10^-/-^ mouse in a related publication,^110^ but this study has not been published independently. Despite these promising findings, CBD has been shown to worsen LPS-induced pulmonary inflammation in mice^111^ and so some caution should be maintained.

Other exocannabinoids which have been beneficial at ameliorating colitis include MFF [an extract of medicinal cannabis],^112^ cannabigerol,^113^ and cannabichromene.^114^

Of all experiments within the published literature assessing modulation of ECS components on experimental ileitis/colitis, few have used non-chemical models. These include IL10^-/-^ colitis [one published study,^73^ and one referenced within the text of another^110^] and TNF^ΔARE^ ileitis.^56^ Several studies have used oil of mustard, croton oil, and LPS to induce colitis [detailed within a recent systematic review^115^] but these models are not classically felt to be experimental models of human IBD, although they may provide insight into intestinal inflammation in general.

Better understanding of the molecular wiring of the endocannabinoid system in the mammalian intestine may enable development of more specific models capable of interrogating the potential protective effects of this pathway via genetic ablation approaches. Further investigation of ECS modulation in other models of IBD and intestinal inflammation, such as *Citrobacter rodentium* and *Helicobacter hepaticus*, would be informative. The development of a first-in-class MGLL inhibitor for human clinical trials, ABX-1431 [Abide Therapeutics^1^], provides opportunity to investigate this in human IBD if preclinical study was supportive.

## 7. Clinical studies and trials in human IBD

**Figure 2.**
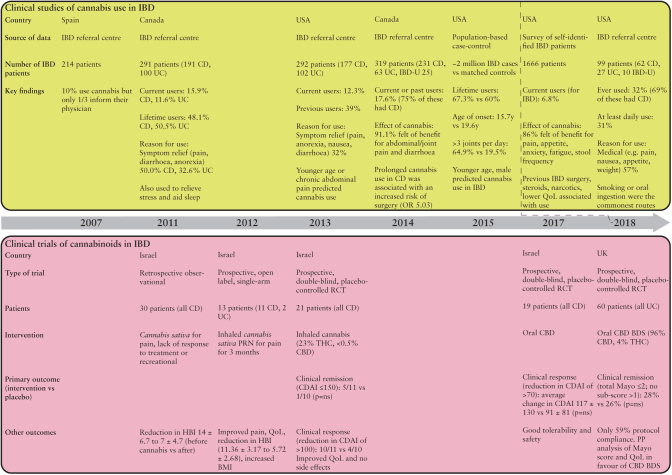
A summary of clinical studies and trials of cannabis and cannabinoids in human IBD. Studies consistently demonstrate use of cannabis in patients with IBD, frequently for symptom relief. As yet, no clinical trials of cannabinoids in IBD have met their primary endpoints but demonstrate improvements in symptoms, quality of life, and clinical severity scores. Data extracted from references 60, 116–124, 126, 128. IBD, inflammatory bowel disease; CD, Crohn’s disease; UC, ulcerative colitis; OR, odds ratio; HBI, Harvey-Bradshaw Index; CDAI, Crohn’s Disease Activity Index; QoL, quality of life; CBD, cannabidiol; CBD BDS, cannabidiol botanical drug substance; THC, tetrahydrocannabinol; PP, per protocol; RCT, randomised-controlled trial; ns, not significant.

Several questionnaire-based studies have confirmed current use of cannabis in 6.8–15.9% of adult patients with IBD, with lifetime use in 48.1–67.3% of patients.^116–120^ A Canadian study found that 17.6% of IBD patients were current or previous users of cannabis specifically for IBD.^60^ Among an adolescent cohort, 32% of patients with IBD had ever used marijuana, 57% for medicinal purposes.^121^ The most common reasons given for cannabis use were to alleviate abdominal pain, diarrhoea, or anorexia^60,117,118,120–122^ and use is higher in patients with previous surgery or chronic analgesic requirements.^117,118,120^ Improvements in quality of life have also been demonstrated^118^ along with a reduction in Harvey-Bradshaw Index.^122^ A large population-based survey confirmed a younger age of onset of cannabis use in patients with IBD and overall heavier consumption.^119^ Another group has identified an association between prolonged cannabis use [>6 months] in CD and a higher incidence of previous surgery (odds ratio 5.030 [95% confidence interval 1.449–17.459]).^60^ The studies are summarised in Figure 2.

An open-label, single-arm study of inhaled THC used ‘as required’ for pain in IBD patients [*n* = 13] demonstrated effects on analgesia, improved quality of life, and increased body mass index.^123^ A reduction in Harvey-Bradshaw Index from 11.36 to 5.72 was seen in patients with CD [11/13] and a slight decrease in partial Mayo score for patients with UC [2/13]. Subsequently a prospective, placebo-controlled trial of inhaled THC [23% THC, <0.5% CBD] in CD failed to meet its primary endpoint of increased clinical remission.^124^ However, effects were seen on clinical response as assessed by the CD Activity Index [CDAI]—a scoring system based on largely clinical parameters with no objective assessment of inflammation.^125^ Patients reported improved quality of life and reduced pain, which likely accounts for the reduction in CDAI without there necessarily being an effect on inflammation. Alternatively, the choice of patients with medically refractory disease in this trial may mask subtle benefits.

The same group performed a double-blind randomised controlled trial [RCT] of oral CBD in CD.^126^ There were no safety issues identified, but they failed to meet the primary endpoint of a reduction in CDAI after 8 weeks. Whereas this may represent a failure of CBD to exert anti-inflammatory effects in human IBD, the randomisation procedure resulted in 6/10 [60%] of those treated with CBD versus 0/9 [0%] of placebo being current smokers. Smoking is well known to be associated with a more difficult-to-treat disease, and this may mask an effect of CBD.

Inhaled *Cannabis sativa* containing THC has been trialled in patients with extensive or left-sided UC refractory to medications, including thiopurines and biological agents, and published in abstract form.^127^ The placebo arm was inhaled *Cannabis sativa* from which THC had been extracted. This demonstrated no effect of THC on C-reactive protein [CRP] or calprotectin levels compared with placebo. A modest effect was seen on disease activity index and Mayo endoscopic subscore [reduction from 2 to 1 in the intervention group, *p* <0.01]. Improvements were seen in terms of abdominal pain, appetite, and general satisfaction, with no clear safety signals.

A double-blind, placebo-controlled, RCT of GW42003 [approximately 96% CBD, 4% THC] in active UC has recently been published.^128^ The trial recruited 60 patients with mild-moderate UC, excluding isolated proctitis, and remains the largest clinical trial of cannabinoids in human IBD to date. Participants were required to be on either no or stable dose of 5-aminosalicylic acid [5ASA] before entry. Importantly, this trial incorporated endoscopic evaluation and measurement of CRP and faecal calprotectin as objective measures of inflammation, alongside clinical scoring. The trial failed to meet the primary endpoint of clinical remission, but a reduction in Mayo score and improvement in quality of life were favoured by GW42003. However, only 59% of those treated complied with protocol, due to adverse effects likely due to the THC component. There is a need for cannabinoids which do not have neuropsychiatric side effects—THCA, discussed earlier, may be beneficial in this regard.

It is interesting to hypothesise why the experimental data are not yet translating into meaningful improvements for patients. This may simply represent immunological differences between species; or that chemical experimental colitis models are insufficient to accurately model human IBD; or that the route of drug administration is wrong; or that the inclusion criteria for patients in some of these trials generally selected for patients with more advanced, and therefore inherently more difficult to treat, disease. Replication of experimental findings in alternative animal models such as T cell transfer or IL10^-/-^/*Helicobacter hepaticus* should be encouraged. Interestingly, a recent study has shown that the Jurkat cell line [T cell line] is more resistant to the effects of CBD when cultured at physiological normoxia [12%] than at 21%.^129^ Given that the intestine is relatively hypoxic,^130^ especially in the context of active inflammation, this may also explain why exocannabinoids have so far failed to live up to expectations from preclinical work.

## 8. Conclusion

Research into the distribution and function of the endocannabinoid system in IBD and models of intestinal inflammation is increasing. There is accumulating evidence that enhancing signalling through cannabinoid receptors 1 and 2 has anti-inflammatory potential in the intestine *in vivo*. This was the subject of a recent systematic review and meta-analysis,^115^ although this paper did not include studies of cannabinoid receptor antagonists and, as mentioned above, did include studies of LPS, oil of mustard, and croton oil-induced colitis. Critically though, this article confirms the bias towards chemical models of colitis. Although cannabis use is fairly common in patients with IBD, particularly to relieve symptoms, the limited number of trials of exocannabinoids in IBD have not met their primary endpoints [Figure 2].

Before novel therapies targeting endocannabinoids, rather than exocannabinoids, can be translated into the clinical setting for IBD, it is essential that sufficient preclinical work is completed. There is an urgent need for better reagents to interrogate the system *in vitro* and *in vivo*. Antibodies are often non-specific,^131,132^ and small molecules do not necessarily target the receptor appropriately,^75^ potentially resulting in misleading results. Many ECS enzymes, including MGLL, FAAH, and DAGL, are serine hydrolases. Activity-based protein profiling [ABPP] can be used not only to profile activity of these enzymes in cells and tissues, but also identify off-target effects of inhibitors on other enzymes within this family,^133^ but has not yet been employed in human IBD.

The development of single-cell techniques opens up the possibility of better understanding ECS tone in individual cells. It is entirely plausible that the ECS functions in the gut in a similar way to the central nervous system where signals are sent between cells to modify neurotransmission.^134^ Improved understanding of the effect of inflammation on the ECS in different cell types might lead to a better understanding of how to translate this into meaningful therapies. At present though, it is difficult to make firm recommendations on the benefit or risk of cannabinoids in the management of the inflammation associated with human IBD.

It should not be overlooked, however, that a beneficial effect of cannabinoids on symptom control in patients with IBD is possible. There are well-documented effects of ECS modulation on gastrointestinal motility. Polymorphisms in FAAH^50^ and CB1^135^ have been associated with subtypes of irritable bowel syndrome in humans, and *in vivo* administration of CB1 antagonists reverses the inhibition of gastrointestinal motility seen with cannabinoid agonists.^136^ Dronabinol, a non-selective cannabinoid agonist, reduces gastric emptying, with a gender bias towards females.^137^ In addition to roles in gastric emptying, cannabinoids [including dronabinol, nabilone, and nabiximols] exert an anti-emetic effect likely mediated through central effects on CB1 and possibly CB2.^138^ The beneficial effects on nausea and vomiting may be lost, however, when cannabis is used chronically—resulting in the cannabis hyperemesis syndrome. This has many features similar to cyclic vomiting syndrome, a condition which has associations with CB1 polymorphisms.^139^ Although the exact mechanisms are poorly understood, it is reproducibly observed that symptoms may be relieved by hot bathing.^140^

The ECS, cannabinoids, and modulation of pain, including in visceral hypersensitivity associated with chronic stress, are inextricably linked and have been the subject of many reviews [including^141^ and^142^]. Indeed, nabiximols [a combination of THC and CBD] is licensed for the treatment of spasticity and spasms in multiple sclerosis, with some effects on pain in this condition.^143^ To this end and with relevance for IBD, a phase 2a clinical trial of olorinab [APD371], a full CB2 agonist, for visceral pain in Crohn’s disease is under way but has not yet reported [ClinicalTrials.gov Identifier: NCT03155945]. Any benefit of cannabis, cannabinoids, and ECS modulation in IBD has to be carefully balanced against the potential myriad negative, including neuropsychiatric, side effects.

Relevant to the rise in addiction to prescribed and illicit opiates, and the associated adverse health outcomes, there are valuable preclinical data suggesting overlap between the endocannabinoid and opioid systems. The MGLL inhibitor MJN110 and morphine act synergistically via μ-opioid and cannabinoid receptors to relieve neuropathic pain, but without the unwanted side effects of reduced gastrointestinal motility and cannabimimetic side effects.^144^ MGLL^-/-^ mice are hypersensitive to the μ-opioid agonist, loperamide^145^ and CB2 agonists have been shown to induce μ-opioid receptor transcription in Jurkat cells.^146^ Modulation of the ECS may increase sensitivity to opioids and therefore may be a strategy to reduce opioid requirements if the evidence translates to human disease.

As calls for medicinal cannabis for treatment of epilepsy and other conditions intensify, it is all the more pressing that we better understand the effects of cannabinoids on human diseases—not just to identify novel applications, whether for symptomatic relief or as anti-inflammatory agents, but also to reduce the risk of exposing our patients to harm.

## Footnotes

1 Abide Therapeutics. http://abidetx.com/news/abide-therapeutics-announces-first-subject-dosed-in-first-in-human-clinical-study-of-abx-1431-an-investigational-endocannabinoid-system-modulator/; Accessed 23 July, 2018.
